# Community and research staff collaboration for development of materials to inform microbicide study participants in Africa

**DOI:** 10.7448/IAS.17.3.19156

**Published:** 2014-09-08

**Authors:** Cynthia Woodsong, John Michael Mutsambi, Smangalisa Ntshele, Peggy Modikoe

**Affiliations:** 1 International Partnership for Microbicides, Silver Spring, MD, USA; 2 Community Engagement Consultant, Cape Town, South Africa; 3 International Partnership for Microbicides, Paarl, South Africa; 4 TB/HIV Care Association, Cape Town, South Africa

**Keywords:** microbicide trials, community collaboration, participant information materials

## Abstract

**Introduction:**

Clinical trials of new vaginal products require careful communication with participants about trial requirements. Most microbicide trials have been multi-site studies conducted among women in sub-Saharan Africa, where literacy levels and understanding of scientific methods differ from those designing and conducting the trials. Microbicide trials require women to insert objects in their vagina and ensure they are present in the vagina during sex. For many women, this is a novel behaviour. These behaviours take place within the context of clinical trial participation, which is an additional novelty. Research teams must develop informational materials to help participants understand the clinical trial and input from local research staff and community members can improve the content and format of these materials.

**Methods:**

This paper discusses the development of illustrated materials developed for microbicide trial participants, presenting examples from two studies. In both studies, research staff and community advisory groups collaborated to review and revise materials.

**Results:**

Collaborative efforts revealed insights about how to convey information about clinical trial participation and microbicide use. These insights highlighted realities of the local context, details that might be misunderstood, illustrations of a sensitive nature and concerns about blood testing. In particular, information about blood testing and product use instructions required careful consideration. Although the research team anticipated needing advice on how best to convey information on these topics to participants, some aspects of potential participant concerns about these topics were also new to the research team. Community advisors and local research staff suggested better ways to convey this information, and provided guidance on how to use the materials.

**Conclusions:**

The collaboration served to develop informational materials for microbicide trial participants. Furthermore, staff gained a better understanding of issues and concerns that could influence trial participation. A collaborative engagement process can provide important insights into local culture and knowledge beyond what is needed for development of clinical trial participant information materials. Research teams should be sensitive to this possibility, avail themselves of information and take appropriate action.

## Introduction

The continued need for HIV prevention methods that women can use provides motivation for efforts to develop vaginal microbicides, which are products inserted in the vagina and meant to remain present during sexual intercourse to impede sexual transmission of HIV [1]. For many women, this is an entirely novel behaviour, and for those accustomed to traditional vaginal practices [2], the specific use requirements for microbicide products may be more exacting. Use requirements have included timing of insertion in association with sexual intercourse, daily routine insertion of gels and insertion of a vaginal ring that is left in the vagina for weeks at a time [3]. These behaviours take place within the context of placebo-controlled clinical trial participation, which is a further novelty, and careful communication is required to support meaningful informed consent and good adherence to study requirements.

Potential study participants, research staff, community members and the family and social network of participants all need clear information about clinical trials conducted locally [4]. Most microbicide trials have included research sites in multiple countries, and research has been concentrated in sub-Saharan Africa, where incidence of HIV infection is high [5]. In this region, study participants’ literacy and knowledge of scientific methods and concepts often differ from those designing and conducting trials and thus special attention is needed to develop culturally appropriate and effective communication materials about trial procedures and requirements. Incorporation of illustrations in educational materials can improve communication [6, 7], and community input is increasingly used in the development of such materials.

The collaborative process for developing communication materials provides insights into issues that influence clinical trial participation and adherence, as well as those that could impact future introduction and use of products and interventions proven effective. In microbicide research, such collaboration usually occurs through interactions with community advisory groups [8–10]. Communication is intended to flow in both directions, from community to the research group conducting the trial, and vice versa [11].

This paper reviews the collaborative and adaptive processes to develop informational materials for participants in two microbicide trials, one initiated in 2005 and one in 2012, and highlights the accumulated experiences with communication needs over that time. The first trial is HPTN 035, a Phase IIB study of vaginal gels conducted by the HPTN at sites in Malawi, South Africa, United States and Zimbabwe from 2005 to 2009 [12]. The second study, initiated in 2012, is IPM (“The Ring Study”), a Phase III safety and efficacy clinical trial testing a vaginal ring and currently being conducted by the International Partnership for Microbicides (IPM) at research sites in South Africa and Uganda [13].

In the 10 years that passed between initiating design of the HPTN 035 materials and completing materials for participants in IPM 027, much had been learned about the issues and communication needs for microbicide trial participants, and this knowledge has been applied to IPM 027. After providing a summary of the types of informational materials developed for trial participants, we discuss the lessons learned through the collaborative process.

## Discussion

Illustrated materials described here were developed for both studies prior to study initiation, so that they would be available for the initial informed consent process. They were not developed using a scientific research protocol, and they were not formally evaluated or tested. Rather, the materials development process began with drafts prepared by a team working at the study coordinating centre (for HPTN 035) and study sponsor headquarters (for IPM 027). This central team then engaged in a consultative process with staff and community advisory groups at the local research sites.

In HPTN 035, local input on drafts of illustrated materials was obtained from staff at the research sites, including staff responsible for community engagement. At some of the sites, these staff in turn asked for informal feedback from members of their local community advisory group. For IPM 027, input was provided by the staff at the research sites and a more formal process was used to solicit feedback from the community advisory groups associated with the research sites conducting the study. Draft materials were initially presented to research site staff and revised according to feedback. Staff then presented the second draft to their community advisory group, and written feedback was provided to the staff member developing the materials at IPM. Materials continued to be revised until the groups involved considered that feedback had been appropriately incorporated. For both studies, translations followed illustration development, using an iterative process that sometimes required additional adjustments to illustrations. Importantly, the community advisory groups in IPM 027 were given formal feedback on how their recommendations were accommodated in the revised and final versions.

Materials were approved by ethics committees prior to being provided to participants. Centralized training on use of all materials developed for both studies was provided to the research site staff, using a training-of-trainers approach. Training manuals included a page-by-page discussion of the purpose and intent of the illustrations, talking points and counselling approaches to be used with the materials. Training was completed and materials in place prior to the start of both trials.

## Types of materials

The educational materials for participants in both trials use illustrations. In both studies, local input was used to develop the main characters for print materials, which included a study participant, her partner and a staff member at the research site. Since both studies included participants from multi-cultural settings, local staff and community advisors advised that the characters be somewhat neutral, avoiding attire and hairstyles that could be identified with a specific ethnic group. For HPTN 035, separate sets of African and U.S. illustrations were developed, although the content and text (translated into local languages) was the same. These characters appeared throughout the sets of illustrated materials, which included the following items:

1. *Informed consent materials*: Both studies developed table-top flipcharts to be used at the research centres during the informed consent discussion, proving information on study design and purpose, procedures, risks and benefits, confidentiality and what is expected of study participants. In HPTN 035, a booklet version of the flipchart was also given to participants to take with them.
2. *Product use instruction sheets*: Both studies developed illustrated instruction sheets to provide participants with instructions about how to correctly use the study products. Instructions for HPTN 035 focused on how to correctly apply the gel before sex and dispose of the applicator. The instructions for IPM 027 carried forward the illustration concepts for insertion, but expanded the instructions to explain what to do if the vaginal ring is out of the vagina between visits.
3. *Blood testing information sheets*: During the period that HPTN 035 was being conducted, experiences were accumulating in HPTN 035 and other microbicide trials, highlighting a number of issues that are challenging to convey to participants [14–16]. These include aspects of blood testing in general and the HIV testing algorithm adopted for the study. In response to this need, an additional set of information sheets was developed for IPM 027, to be used as needed throughout the trial.

## Adapting to local context

Some of the required revisions were fairly straightforward and would likely have become apparent in a routine review. For example, community advisors noted that not all have access to running water, and thus an illustration of product rinsing was changed to convey a basin of water, rather than a spigot. Similarly, the community advisory group in one country noted that motorcycle transportation is much more common in their area than the minibus portrayed in the original illustration, and thus a motorbike was added to the page. Although both of these example revisions were easily accommodated, it is worth noting that the discussion which highlighted the local context served to further sensitize the research staff across all sites.

Within the local context, some specific items to be illustrated revealed sensitivities of a personal nature. For example, community advisory group members objected to one of the initial depictions of vaginal ring insertion. The instructions show options for inserting a ring either by squatting, lying down or standing with one foot raised. Some community members felt that one of these illustrations could be misconstrued as portraying masturbation. The illustration for IPM 027 was easily modified to depict the ring clearly in the participant's hand. The HPTN 035 informed consent flipchart included an illustration of a woman receiving a pelvic exam, and local advice was to not include this in the booklet version given to women to take home, since it could be embarrassing or confusing if seen by children.

## Clarification of instructions for correct use of microbicides

The product use instructions developed for both studies are undoubtedly stronger because of the local input received. The information is more in tune with the realities of participants’ lives and possible misperceptions have been clarified. The instructions provide a convenient and appropriate visual aid to reinforce product use-adherence. For example, the IPM 027 product use instruction sheet makes it clear that sometimes an expelled ring should not be reinserted, while in other cases it can be rinsed and reinserted. The sheet depicts only ring insertion, not removal, in order to avoid conveying the message that the ring should be removed. The product use instruction sheet used in HPTN 035 clearly illustrated that the applicator should be inserted only halfway into the vagina; this was meant help avoid a mistaken assumption that women should strive to push the applicator in as far as possible, which could possibly cause trauma.

## Addressing blood testing concerns

An important issue that has given rise to rumours and misperceptions in HIV prevention research, including microbicide trials, concerns blood testing [14–16]. In microbicide trials, routine and repeated blood testing is required over extended periods of time (e.g. 1 to 2 years or more), and some scheduled tests may collect multiple vials of blood. The volume and/or frequency of blood testing has given rise to rumours that researchers sell blood for profit-driven or nefarious purposes, including “Satanism.” Some who accept explanations about the need for and purpose of blood testing may nevertheless be concerned about what happens to the blood once tests are complete (e.g. Is it stored? Is it sold? If it is destroyed, how is that done?). On a different note, staff at some research sites noted that participants may not understand how quickly blood is replaced in the body, or how much blood the body contains. Finally, those working in the larger field of HIV/AIDS have long reported that people find HIV testing algorithms confusing, and they may not believe the accuracy of testing results.

**Figure 1:**
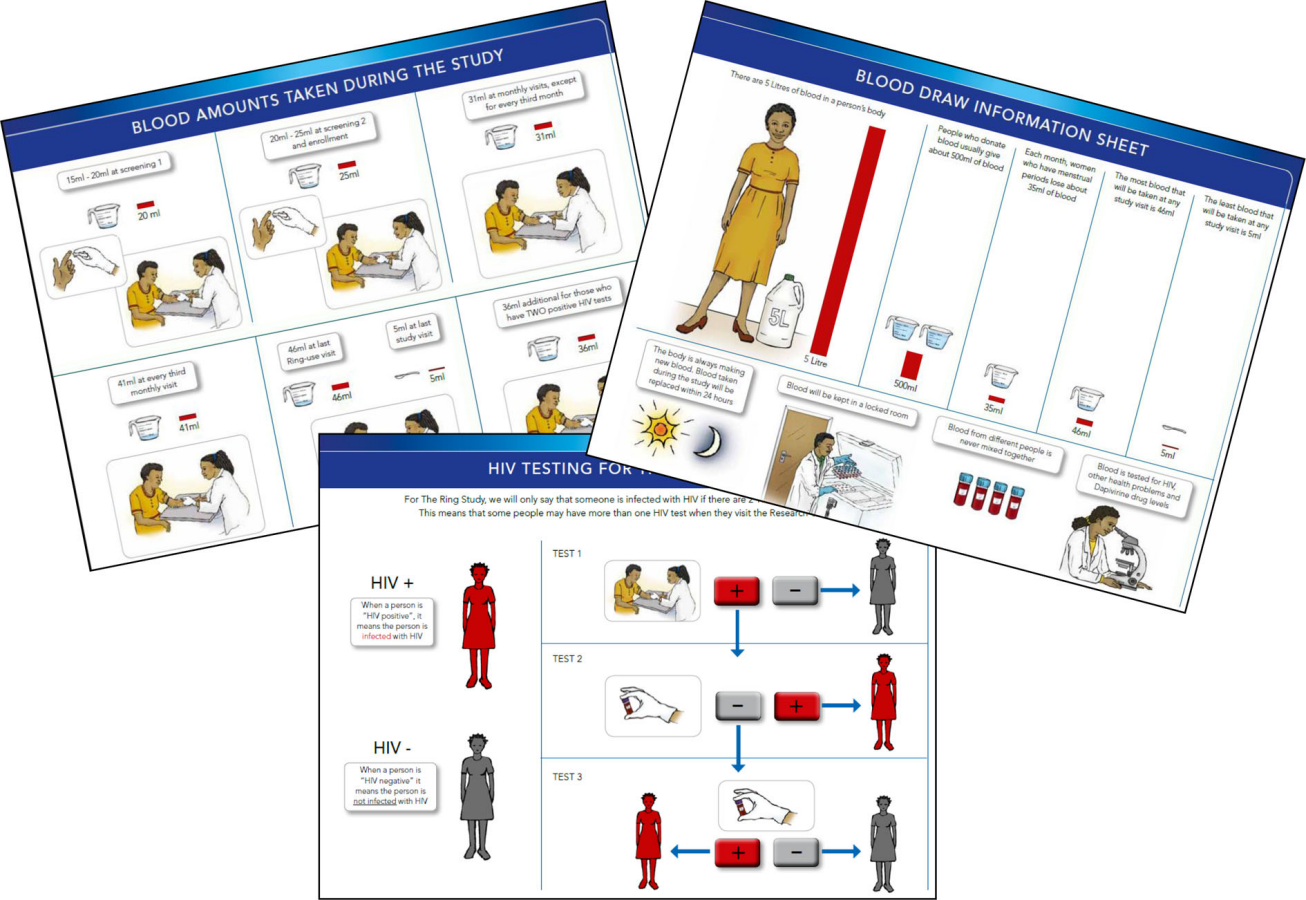
Supplemental information sheets about blood testing.

On the advice of local staff, the HPTN 035 illustrated materials depicted a hand holding a blood collection vial, to counter previous rumours that cola-sized bottles of blood were collected. For IPM 027 three illustrated sheets were developed to explicitly address the multiple blood draw issues that had been raised in previous trials, and by the community advisors and staff working in the trial sites (Figure 1). One sheet is a simple diagram of the HIV algorithm, using illustrations and characters from the informed consent flipchart to show the sequence of testing when an initial test is positive. A second sheet illustrates the amount of blood drawn at different types of visits (e.g. monthly, quarterly) throughout the trial, using common household items, such as a spoon or measuring cup to convey volume. This sheet is available to supplement the informed consent discussion, as well as at any visit when blood is taken, or the participant expresses concerns or questions. A third illustrated sheet shows how much blood is in the average women's body, again using a household item for comparison, and compares that volume with the amounts taken during the different study visits as well as how much blood a woman normally loses during menses. This sheet also explains that the body replenishes blood within 24 hours, samples are kept in a locked location and that blood is subjected to tests for “HIV, other health problems and dapivirine drug levels.”

Development of all these materials benefitted from community consultation. For example, it was requested that the algorithm sequence, which uses illustrated silhouettes, be accompanied by the flipchart illustration of a blood draw, which had previously been scrutinized and revised to meet community approval. The household items to be used for comparison with various blood volumes were discussed and agreed upon. At the community advisors’ suggestion, an illustration was added to explain that a participant's blood is never mixed with anyone else's. This concern of participants was previously unknown to the research team developing the illustrated materials. The research team was unable to develop a satisfactory illustration of how blood is destroyed (illustrations depicting burning were considered too alarming), so this concern was added to the talking points only.

## Conclusions

The use of illustrations has been shown to be an effective way to convey health information, particularly in low-literacy settings [10]. It is incumbent on research teams to make such materials available to the larger research field, so they can be adapted through a consultative process for use in other settings. Indeed, over the time period covered during the two trials discussed, the basic style for illustrated consent materials and user instructions described in this paper has become widely used in microbicide trials. The blood-testing information sheets are unique, and they provide an example of how research teams with a foundation of illustrated materials can develop additional materials as needed throughout a study, building on participant familiarity with the characters and information format. Although communication about clinical trial objectives, procedures, requirements and risks and benefits is particularly concentrated during the enrolment process, such communication is needed throughout the study period [4].

As an added benefit, the review and consultation process provided the research staff and community advisory groups with a deeper understanding of the trial procedures and requirements, and community concerns and interests. The discussions helped elucidate issues known to be problematic, and surface new ones that required the attention of the research staff. Moving forward, future clinical research could continue to benefit from the accumulated lessons learned through collaborative communication with local research teams and community advisory groups. Knowledge of the experiences of trial participants, the rumours that circulate in trial communities about blood testing and continued engagement with community advisors will be helpful in future research as well as introduction of HIV prevention methods.
